# “I Could Not Manage This Long-Term, Absolutely Not.” Aging in Place, Informal Care, COVID-19, and the Neighborhood in Flanders (Belgium)

**DOI:** 10.3390/ijerph18126482

**Published:** 2021-06-16

**Authors:** Jakob D’herde, Wesley Gruijthuijsen, Dominique Vanneste, Veerle Draulans, Hilde Heynen

**Affiliations:** 1 Department of Architecture, KU Leuven, 3001 Heverlee, Belgium; hilde.heynen@kuleuven.be; 2 Department of Earth and Environmental Sciences, KU Leuven, 3001 Heverlee, Belgium; dominique.vanneste@kuleuven.be; 3 Centre for Sociological Research, KU Leuven, 3000 Leuven, Belgium; veerle.draulans@kuleuven.be

**Keywords:** aging in place, informal care, COVID-19, neighborhood

## Abstract

Public health and care policies across OECD (Organisation for Economic Co-operation and Development) countries increasingly encourage aging in place, enabled by both formal care networks, and informal (family) care and social solidarity in the neighborhood. However, little is known about how a person’s neighborhood might affect their aging in place. The COVID-19 crisis unintendedly offered a good opportunity to observe the neighborhood’s role in the provision of care. Since formal care services were often limited during the lockdown, informal caregiving may have increased. However, intergenerational contacts in and outside of the household were strongly discouraged by governments worldwide, adding another layer of complexity to caregiving. The aim of this qualitative study was to assess how informal caregivers in Flanders managed to provide care to their care receivers, and what role the neighborhood played in this provision of care. Sixteen qualitative Skype and telephone interviews with informal caregivers were conducted between June and December 2020 to understand their experiences and coping strategies. Overall, most respondents increased their frequency of caregiving during the first lockdown. They took on the extra care needs during the lockdown themselves, and did not actively invoke any kind of neighborhood support. The significance of the neighborhood seemingly remained limited. This was often not because no help was offered, but rather due to a sense of pride or the fear of infection, and an increased effort by family caregivers.

## 1. Introduction

The COVID-19 pandemic had a major impact on people’s lives worldwide. Like in many other European countries, most people were confined to their own home during the first lockdown in Flanders (Dutch-speaking region of Belgium), which lasted from 18 March to 3 May 2020. Supermarkets, pet food stores, and pharmacies were considered essential and remained open, although older people and people at risk were discouraged from going there themselves. Residential care facilities were closed for visitors, as were local service centers offering activities and support to older people [1]. Contacts with older people were discouraged, both inside residential care homes and in their own dwellings or environments, as illustrated by a statement from Wouter Beke, the Flemish minister of Welfare, Public Health, Family and Poverty Reduction: “The message should be clear: stay away from older and frail persons, unless there are no alternatives. [...] This not only applies to residential care settings, but to the whole society” [2], translated. Even informal care was discouraged when it was not absolutely necessary [3]. However, to what extent informal care could be considered as essential was not very clear or specified. The general message was that grocery deliveries were essential, but having a chat was not. Hale, Barrett, and Gauld [4] have pointed out this issue over a decade ago, arguing that because social contact is generally not considered an essential activity, older people experience barriers to ask for assistance to meet their social needs. Care is often narrowly understood as a set of physical tasks, instead of as an entire range of activities, including social interactions. Our own understanding of care adopts the latter, more inclusive viewpoint [5]. The authors acknowledge that there are equally numerous ways to define caregivers. In this article, informal caregivers are defined as friends, neighbors, and family members who provide care to an older adult. They may receive some compensation for this care (such as financial governmental support when registered as a caregiver), but it is not their professional job. 

**Figure 1:**
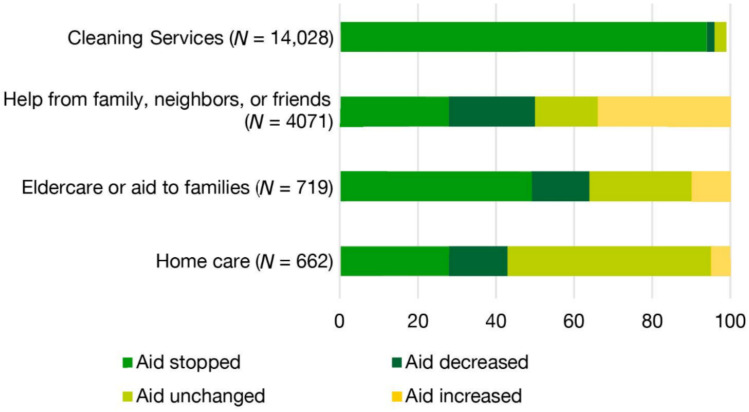
Percentual changes in care provision in April 2020. The first weeks of lockdown saw major decreases in care provision, both for formal and informal care, despite a concurrent increase in informal care for some. The image is reproduced and translated from [7].

As movements were highly restricted both nationally and internationally, people were confined to their home and immediate neighborhood. What did this mean for the patterns of informal care? Older people might have had to appeal more to their family, but also to their neighborhood networks to obtain daily necessities, since they were discouraged from going to the supermarket themselves. Older people without family caregivers living nearby might have become more dependent on the neighborhood, whereas older people without any informal family care apparently were not taken into account in the initial decision-making process (though many cities did call older residents and started online platforms where citizens could ask for help [6]). Moreover, during the lockdown, considerable amounts of formal and professional care were often cancelled or drastically limited, further increasing the pressure on informal care ([7], see Figure 1 for Belgium). Such lockdowns happened in most countries, and their effects on informal care provision are currently being investigated. On the one hand, it is clear, as Chan et al. [8] mention, that “[d]uring large-scale public health emergencies, home care may be the only viable method of providing continuous healthcare”; on the other hand, there is a need for more in-depth knowledge about informal home care and informal home care providers and their involvement (family or neighbors). Quantitative studies from Germany and Austria reported that the psychosocial burden on many informal caregivers intensified during the pandemic [9,10]. Qualitative studies in Serbia and the USA likewise found an impact on the mental health of informal caregivers [11,12]. This paper adds to this qualitative body of knowledge, focusing on the experience and coping mechanisms of informal caregivers in Flanders during the COVID-19 pandemic. We were especially interested in how informal caregivers managed to provide care during this challenging time, and what role the local neighborhood and other (informal) networks played. Thereby, we complement large-scale quantitative studies, such as the ones from the federal research institute Sciensano [7]. 

### 1.1. Aging in Place: The Neighborhood as the Missing Link

In Belgium, as in most other OECD (Organisation for Economic Co-operation and Development) countries, eldercare policies stimulate aging in place, often narrowly understood as getting older in one’s own family dwelling. Concurrently, policies increasingly focus on the socialization of care, whereby care is seen as a shared responsibility [13,14]. In reality, this means an increased focus on informal care, which is often combined with a decentralization or re-scaling of governmental care responsibilities [15,16]. 

Remaining at home is the wish of most older people themselves, expressed while still in good health. Many associate home with positive memories, mostly at times they had no health restrictions [17]. Moreover, the fear of moving and losing their social network is an important factor [18,19]. Another reason for this general preference can be the lack of attractive dwelling alternatives. Alders and Schut [15] indeed mention that residential care facilities suffer from negative connotations (also because their population dominantly consists of older people with severe physical or mental constraints), while other alternatives are lacking, not well-known, or complicated by administrative rules. However, many authors question the desirability of aging in place, refuting its apparent reputation of being the best option from a health and macro-financial perspective [20,21,22], and when it comes to care supply [15].

Due to the narrow focus on the home, policies often neglect the fact that they implicitly assume the availability of a supportive family, neighborhood, and environment [23]. Unfortunately, a comprehensive viewpoint on aging in place is often lacking [24]. Although there are initiatives, such as the WHO Age-Friendly Environments, little is known about what neighborhood characteristics contribute to the enabling of aging in place. Even when it comes to environmental gerontology and the so-called person–environment fit, the focus is predominantly on the dwelling itself, while the role of the dwelling’s immediate environment—the neighborhood—is neglected [25,26]. We agree, therefore, with authors who argue that more attention is needed for the meso-level, or more specifically, the level of the neighborhood [27,28]. In the context of the pandemic, we define the neighborhood as the living environment in which people were allowed to move freely during the lockdown. 

### 1.2. Informal Care during the COVID-19 Pandemic

Whereas the aging in place policy is based on the idea that informal care will be able to cover a lot of older people’s needs, several international studies have questioned the availability of informal care due to demographic and societal shifts [11,29]. In general, the number of potential caregivers for the oldest age groups is decreasing, because the oldest cohorts continue to grow faster than the generations following them [30,31]. Besides, informal caregiving is not without costs itself, and can impact the quality of life and wellbeing of the caregiver [32]. Providing care impacts labor force participation, particularly for women, who still provide the bulk of informal care [33]. Moreover, the extent to which care is seen as a responsibility of the government on the one hand or family on the other differs between countries and cultures [34], and an increased focus on informal care does not necessarily mean that informal caregiving will, or even can, increase [35]. Some people do not want to shift their care burden to their children, and mainly consider the government and care institutions responsible to provide care [36], while others (e.g., family members) consider the opportunity costs to provide care too high [37,38]. 

All of these factors became even more evident during the pandemic’s first lockdown. Reports of the federal research institute Sciensano show that in April 2020, almost 50% of Belgian older people making use of formal home care (provided at their individual dwelling) did not receive this care during the first weeks of lockdown, while 94% of people using cleaning services noticed their cancellation ([7], see Figure 1). This implies that the gap in care had to be filled somehow. Whereas more than 35% of the report’s respondents indicated that the help of family, neighbors, and friends increased during this period, half of them reported a decrease or full stop in informal help. Clearly, the hypothesis of informal help becoming more important during the lockdown needs nuancing. However, Sciensano’s research does not differentiate between family, neighbors, and friends as informal caregivers [7]. Hence, our research is complementary, because it specifically looks at support from the neighborhood as an alternative for informal family caregivers. 

## 2. Research Goals and Methodology

This paper is a part of a larger research project on older people aging in place and the role of distance in the negotiation of care, which was approved by the KU Leuven ethical commission in 2018. Its relevance has been significantly highlighted by the COVID-19 pandemic. To explore whether the dynamics of informal care patterns changed—and, more specifically, whether the appeal to the neighborhood increased—a qualitative methodology was set up. Since it happened to be impossible to reach out to old age care receivers themselves, 16 in-depth interviews were conducted with informal caregivers across Flanders. Participants were recruited through personal connections of the authors and consequently through snowball sampling, with the inclusion criterion of providing informal care. All were contacted via telephone or email after expressing interest in participating in the research. All respondents gave their informed consent with anonymity guaranteed under all circumstances for themselves and the people they talked about. A brief explanation of the research was given both in the first contact and at the beginning of each interview. The participants were all invited to ask any questions they had at the end of the conversation. In total, 16 respondents were interviewed between June 2020 and December 2020 by the shared first authors. The average duration of a conversation was 37 minutes, and the shortest and longest calls took 17 and 79 minutes, respectively. Because our primary respondent sample came from within our circle of acquaintances, we have an overrepresentation of higher-educated persons. The study design aimed to gain in-depth insight on how informal caregivers coped with their responsibilities, and whether they made use of additional support from the neighborhood. Therefore, a qualitative approach was adopted, which was more appropriate to gain in-depth insight into the coping strategies of informal caregivers [39] or perceptions of caregiving itself [40]. Respondents came from both suburban and urban localities, with a small overrepresentation of (smaller) urban areas. As this is an exploratory study, the limited number and specific profile of the respondents were acceptable, given the data saturation. Seven calls were conducted by telephone, nine using Skype, twice without a video presence from the respondent, and seven enabled by video. Moreover, through snowball sampling, we interviewed one member of a neighborhood initiative, whom we questioned about the demand and offer of neighborly help. 

During the interview, participants were asked to reflect on how they contributed to the care provision of the older people they assisted. A literature-inspired topic guide was agreed on to facilitate free conversation flow with two key topics in mind: care and distance, and the meaning of the neighborhood. This paper focuses mainly on the latter topic. The final guide included these preconceived items:

1. The state of affairs before the lockdown: neighborhood characteristics and networks, living arrangements, role assignments, etc.
2. The impact of lockdown on the care provision: appeal to the neighborhood, shifting caregiving roles and intensities, non-essential movements, increased burdens, etc.

The thematic analysis followed the six steps proposed by Braun and Clarke [41,42,43]. The analysis did not follow a linear process, but rather consisted of several iterations of and jumping between these six steps. The interviews were transcribed verbatim (except for two conversations), including non-verbal utterances (like sighs and pauses). All records of raw data were archived as anonymized recordings and verbatim transcriptions. During interactive weekly meetings, the research team decided on five major themes, namely care, home, neighborhood, social contact, and distance. Starting from these broad predefined themes, the shared first authors independently coded the interviews selectively, openly, and axially [40] using the qualitative analysis program QDA Miner Lite. Each resulting code was subdivided into three subcategories: before, during, and after lockdown.

### 2.1. General Profile of Respondents

#### 2.1.1. Caregivers

Due to the COVID-19 safety measures and difficulties interviewing older people digitally, we interviewed their caregivers instead. All but one of the caregivers (*N* = 15) interviewed were between 50 and 69 years old and were an adult child (or child-in-law) or family member of the care receiver(s). All but two respondents were women (*N* = 14). A small majority (*N* = 9) provided care for only one older person, usually their mother (*N* = 6). Only one participant explicitly provided care to non-family members in her neighborhood. Ten caregivers were married, three did not divulge this information, and three were divorced, widowed, and unmarried, respectively. Seven caregivers provided care to multiple persons, either several family members or neighbors. Most caregivers (*N* = 13) were not alone in the care provision and could count on others, usually siblings. Nevertheless, three quarters of our interviewees explicitly stated that they took on the highest care loads. 

#### 2.1.2. Care Receivers

Most care receivers were older than 80, living at home, and a parent (or parent-in-law) of the caregiver. On average, the older persons had received care for about nine years. A minority of care receivers had recently moved (*N* = 7), the majority had lived in their current house for over 30 years, and one lived in a residential care facility. 

#### 2.1.3. Caregiving Characteristics

More than half of the respondents lived in urbanized or semi-urbanized areas close to the people they provided care for; two cases showed co-residency between the adult children and parents, and another two had a formal caregiver living with their care receiver coming from Eastern Europe. Caring needs ranged from nearly independent to high-care needs, with some (*N* = 7) needing medium to high care. In a slight majority of cases, the main care receiver was still able to leave their home, either with help or independently (*N* = 9). In a quarter of the cases, one or more inhabitants never left their home due to high caring needs. The vast majority of cases (*N* = 11) made use of some type of formal care other than cleaning services, such as homecare or family aid. Before the lockdown, all participants provided care on a weekly basis, with almost half doing so several days a week and a quarter daily. During lockdown, these numbers increased; half of the respondents reported providing care daily and a quarter several days a week. There was only one decrease in frequency: an older lady living in a residential care facility could not receive her caregiver, as visits were strictly forbidden throughout the lockdown.

### 2.2. Ethical Approval

Ethics approval for the research project was given by the KULeuven SMEC review board (social and societal ethics committee) on 30 October 2018, and filed under G-2018 10 1355.

## 3. Results

Our results showed the pandemic’s impact on habitual caring practices and older people’s neighborly connectedness. Firstly, we explain how our respondents dealt with the pandemic; secondly, how caring responsibilities shifted; and thirdly, what role the neighborhood played within that transition.

### 3.1. Dealing with the Pandemic

Our findings indicated that a substantial amount of formal care was cancelled or limited due to the COVID-19 pandemic, and often compensated for by existing informal family networks, mostly by the primary family caregiver. Moreover, family caregivers did not seem inclined to call upon neighbors or the neighborhood to help in the care provision. Some even discouraged their care receivers from accepting such help when offered, possibly out of fear of infection. In general, we noticed that social and support networks shifted significantly during the lockdown, often related to governmental mobility restrictions restricting non-essential movements. However, people coped with these rules differently, as they found ways to keep in touch and to negotiate both physical and emotional distance. According to our respondents, some older persons insisted on their informal caregivers, both family and friends, entering the house. Others asked family caregivers not to enter their home and to drop off groceries in the garage or at the front door. Furthermore, some caregivers visited their parents multiple times a week as they did before the pandemic, while others developed a strict rotation in taking turns. In some families, this led to frustrations, for example, when one child wanted to limit contact with his/her parents while the sibling(s) objected. Additionally, several care receivers, especially those less mobile and homebound, were reportedly not always aware of the reality of the pandemic, and made unrealistic demands in terms of specific goods and services to provide. 

In general, for many people, the neighborhood likely became more important due to mobility restrictions (the media reported, for example, about neighbors applauding medical staff together, each on their own doorstep, every night at 8 pm during the first lockdown). We expected that this increased importance of the neighborhood might also have played a role in the provision of informal care. However, according to our respondents, the neighborhood did not become a larger source of informal care, and, in most cases, the appeal to the neighborhood remained limited, as was the situation before the start of the pandemic. This may partly be a consequence of the older persons’ health conditions, as many struggled with mobility and were therefore largely confined to the home even before the pandemic, having limited contacts with neighbors and within the neighborhood anyway. However, for those who still were mobile, the neighborhood did not automatically become more important in daily life. In some cases, informal caregivers restricted or strongly discouraged older people from going out during the first weeks of the lockdown, potentially disrupting already limited social networks. Moving to stay somewhere else also was not seen as a viable option. Many of our respondents noticed that their care receivers had the explicit wish to remain in their own dwelling. Due to the large number of COVID-19 outbreaks in residential care facilities and ill treatment of the crisis at these facilities during the first wave [44,45], many older people’s distrust towards residential care facilities was reinforced, and most respondents supported their care receivers’ wish to stay put even more. 

### 3.2. Shifting Responsibilities between Formal and Informal Care: Negotiating Care Giving 

#### 3.2.1. Taking Over Formal Care

Before the outbreak of the COVID-19 pandemic, most of our respondents indicated that their care receivers made use of some formal home care or formal services, mostly for bodily needs and household tasks, such as cleaning or ironing. During the first weeks of the strict lockdown, access to formal home care was, however, limited. In several cases, formal services and care were cancelled by the providers, which resulted in informal family caregivers taking over these tasks due to the lack of alternatives. However, even when formal caregivers were still willing to provide care, some of our respondents reported purposefully cancelling habitual formal caregivers out of precaution and taking over the tasks previously performed by those formal caregivers. In one case, the informal caregiver not only cancelled all formal home care, but also decided to move in with her parents to co-reside for the duration of the lockdown and to provide the necessary help. As an only child being able to work remotely from home and living in a different city than her parents, she considered this to be the only viable option:

“So also the cleaning lady did not come anymore. More specifically, it meant that all these tasks [doing groceries, light medical care, cleaning the house] […] ended up with me. So that’s why I moved into their bubble [temporarily moved in their home] because otherwise, that would’ve been impossible to manage.”

Adult daughter, ±20 km, Heverlee—Tienen

Most care receivers were very willing to accept formal help again when the situation improved, often because these professionals were recognized as important social contacts as well. However, sometimes caregivers were more reluctant, and postponed formal care a few more weeks or even months. The main reason was a fear of infection related to a certain level of distrust regarding the formal caregivers disobeying the rules and/or not wearing masks (which was not yet mandatory during the first lockdown). This was not always thoroughly discussed with the care receivers, creating tensions when they were close to their formal caregivers. Other factors impacted this decision-making process too, as one respondent indicated that she wanted to prevent her parents ending up in the hospital at all costs, completely isolated due to visitor restrictions in hospitals:

“I’m truly very scared that—imagine they fall and it’s actually a broken leg that you could easily fix, but they go to the hospital and they can’t see anyone for a week over there, yes, that’s just horrible for them.”

Adult daughter, ±10 km, Linden—Heverlee

#### 3.2.2. New Caregiving Roles: Negotiating Emotional and Social Distance

Most respondents took up a higher caring load to compensate for the loss of formal care services. Their motivation to care was high, though some felt their boundaries of intimacy being crossed, especially when it came to more hygienic tasks that were not performed before the lockdown. One respondent summarized this very well by indicating that, even though the care itself became much less spontaneous in general, becoming more formal, distant, and fixed in time, at the same time, it became more intimate:

“I have never been very intimate [with my mother]. But, on the one hand, additional intimacies were added, as I for example had to help my mother going to the toilet, when keeping distance [1,5] meter is impossible, while on the other hand, it is much less intimate, exactly because of corona. That’s sometimes difficult. Particularly in the first weeks, we worried, when we had some contacts, like I hope I don’t have anything [COVID-19].”

Adult son, same neighborhood, Brasschaat

For some, the extra care burden generated a lot of stress as well. Some of our participants suffered a loss during this period, generating even more complex situations of care negotiating. To one participant, it became apparent that similar situations were not tenable long-term; caring became a full-time occupation, profoundly disrupting her life:

“I truly have noticed that this, I could not manage this long-term, absolutely not. This is unsustainable, also because, well, I have a very active life, right? This is truly ending up again in a situation where you really become, well, the nurse in fact, the. . . housewife let’s say.”

Adult daughter, co-residence, Oostende

Several participants used a rotation system to share responsibilities related to care and help, both before the pandemic and during the lockdown. The difference before and during the lockdown was that these visits and rotation systems generally became more formal, as a spontaneous visit to the older parents became less likely. Interestingly, the sibling who already took on most of the care duties before the pandemic kept doing this during the lockdown in most cases, often to a higher degree:

“Honestly, not much has changed. I might have three brothers, but they don’t really show up anymore. Exactly because I’m now in a bubble of five with my sister and my mother. Also, all three of my brothers work and don’t really visit to give care.”

Adult daughter, ±12 km, Brasschaat—Wijnegem

#### 3.2.3. Negotiation Distance in Caregiving 

In most cases, the rotation systems worked, although they could sometimes lead to light tensions or arguments, in which one of the siblings did not want to visit too frequently and insisted on keeping distance with the parent, for example, by only visiting in the garden. While most respondents lived close to the care receiver, geographical distance influenced the caregiving process. Oftentimes, living closer to the care receiver was exactly the reason someone became the primary caregiver, both before and during the pandemic. In other cases, geographical distance became an important limitation to visit, especially when one of the siblings was living abroad and was not able to visit and/or temporarily relieve the pressure on the primary informal caregiver. Even when multiple siblings lived in the same municipality and therefore were close by, geographical distance played a role, in the sense that, for example, one of the siblings passed by the home of his parents daily, making him the one paying spontaneous visits. In another case, the care was equally distributed between siblings, but the one living closest to their parents was responsible for emergency situations or urgent matters. The consistency of this process indicated a shifting of duties to the sibling living closest after a residential move of the previous habitual caregiver. 

In combination with mobility restrictions, we expected that distance would become a larger threshold to help in times of the pandemic, but it seems this was not necessarily the case for our respondents. Nevertheless, in some cases, the mobility restrictions were mentioned, in which it was emphasized that it was not always clear if providing help and care was classified as an essential trip by the authorities. Sometimes, this led to frustrations. One of our respondents indicated that she and her sister lived 75 km away from their mother, and that the restrictions complicated the care provision. Moreover, since their mother was living in a very rural area without neighbors close by, neighborly care was not an option. Therefore, she and her sister kept visiting once a week to bring groceries and other necessities. Furthermore, she argued that social contact was just as important as the actual caregiving, expressing her frustration that her mother could not see her grandchildren:

“Plus, that’s not acceptable, that a grandchild makes a non-essential trip of that many kilometers, right? Because that really bothered me, that so-called non-essential trip, because those aren’t unnecessary trips to us, you see?”

Adult daughter, ±75 km, Diependaal—Bree

Even in co-residence, with distances at a micro-scale, adjustments had to be made. One of our respondents living in an intergenerational residential unit explained how he and his wife avoided the elevator and took the stairs only, so that his parents-in-law were the single users of the elevator, and agreed on a schedule for using the shared spaces, like the laundry room. A secondary schoolteacher living in the same street as her parents indicated that, while she had more flexibility in visiting her parents, at the same time, the combination was sometimes more challenging than expected due to extra (digital) work. In other words, a closer distance to the care receiver might not automatically lead to a better situation, and could even disrupt certain patterns.

#### 3.2.4. Negotiating Care Receiver Expectations

In everyday life, expectations regarding how the people one encounters will behave or react are what structures human interactions. This is even more the case in long-lasting relationships, such as parents–adult children relations. Sometimes, parents who are in need of formal or informal care express difficult expectations towards (one of) the children. COVID-19 urged for flexibility; care receiver expectations sometimes needed to be adjusted. The fear of transmitting a potential COVID-19 infection, or even losing a loved one due to COVID-19, was reflected in several interviews as a factor that mentally impacted the informal caregivers. In one family, the rotation system was frequently changed after someone was afraid to have too many contacts in a specific week. Others mentioned that they did not change the frequency of care, but they kept distance at all times. In another case, the respondent delayed medical care for her mother during the weekend. Since the personal general practitioner was unavailable and she would not trust an unknown doctor, the doubt burdened her heavily. She decided to wait one more day, because of the fear that her mother would have been hospitalized and all alone in the hospital, where visits were heavily restricted. Another factor sometimes complicating or even affecting the informal care or help was that care receivers did not always grasp the situation in the outside world, expressing unrealistic desires during the first lockdown and expecting their informal caregivers to cater to their exact needs. Different respondents illustrated their story through grocery shopping: specific products had to be bought in one particular shop rather than in another. Clearly, some had troubles in dialing back their expectations for the time being:

“What I considered as the most frustrating is that mama didn’t know about the situation at the time, when people were hoarding groceries. And how creepy it was to do groceries. And she kept asking groceries, from this . . . and that . . . And yes, these items were not there. And there was such a grim atmosphere in the store. But she kept acting normal. And of course she also didn’t really know what was going on.”

Adult daughter, same street, Lokeren

### 3.3. Families Versus the Neighborhood: Lack of Support from and Appeal to the Neighborhood 

As mentioned before, current health policy assigns an increasingly important role to informal care, including care from neighbors. However, the COVID-19 pandemic showed that people might be reluctant to rely on informal help from neighbors. Our findings suggested that the neighborhood’s appointed role is not wholly realistic, at least not for older people with family caregivers. As one of our respondents remarked, there is a general expectation that health services should be formally provided, rather than informally through the neighborhood network, when children or family cannot cope with a problem:

“If we got ill ourselves, we’d probably use the health insurance funds or real medical services, not the neighbors.”

Adult son, co-residence, Ghent

#### 3.3.1. Appeal to Neighborly Help

In most cases, there was no active pre-COVID neighborhood community network that organized informal help and support, or at least not one that the respondents were aware of. Still, we must note that this may be due to our secondary source information; older people themselves might experience small neighborly connectedness without their caregivers’ awareness. In a few cases, our respondents mentioned the yearly organization of a neighborhood party or some volunteering. During the lockdown and throughout the pandemic, several new initiatives emerged to help older and vulnerable people. Some were initiated by local municipalities, and thus were organized by local authorities, while others were organized more bottom-up by locals, neighborhood networks, or volunteering groups. Whereas neighborly contacts increased (e.g., through the daily applause already mentioned), this does not seem to have led to a higher level of shared informal care. In the view of our respondents, the willingness to help might have been there on the part of the neighbors, but the older people themselves were very hesitant to take them up on the offer. Most respondents indicated that there was no need to appeal to the neighbors or the neighborhood, because the family network could cope with the increase in caring needs:

“They put little notes in the mailbox as well to ask ‘look, do you need help?’ So that happened. But I mean, the family network managed, so you don’t appeal to those notes. But I suspect that if you ask the neighbors, people would agree to help.”

Adult daughter, same street, Lokeren

Of course, these neighborhood initiatives might have been (and may still be) important for older people without family networks. Nevertheless, a member of such a neighborhood initiative noticed that, in general, not much response followed after putting flyers offering help in the mailboxes. Though many people offered help (including inhabitants that previously never joined neighborhood activities), barely any request for help was made. In one case, an older lady asked for some groceries, but cancelled this request after her daughter committed to do this the next day. Another respondent was connected to the local parish, apart from providing care to her father. The parish called their parishioners to see if they could help them; reportedly, no practical help was needed. Another respondent mentioned that she had asked for help with groceries once, but that no systematic requests were being made. Participants already active in neighborhood initiatives usually stayed active in the lockdown, but had often shifted to more digital means. The respondent who took up care for several neighbors mostly helped them with digital matters, like ordering adult diapers online. Only on two occasions did she report taking on other caring tasks:

“I do the same as before corona. But sometimes a little extra, when that professional care falls through, that you intervene. I cleaned once and I washed someone’s hair once, which I normally never do.”

Neighbor, same neighborhood, Ghent

#### 3.3.2. A Matter of Trust?

In general, people did not want to appeal to neighbors and the neighborhood, both before and during the lockdown. One reason is that they though that they should not bother neighbors with personal or family care-related matters. This was a feeling that was shared by both care receivers and caregivers:

“But it is true that the neighbors say . . . if we can do something . . . and so on, but yes, you cannot burden neighbors with providing care for an older person. It’s not that I can never give a call like, I have a problem . . . but most of the time the problem is of medical or caring nature, whereby I can for example call the pharmacy instead.”

Adult daughter, co-residence, Oostende

This general unease to ask for help was not only related to asking for bodily care needs, but also for other forms of help, like shopping for groceries or doing home repairs. Nevertheless, respondents expressed being sure that they could call their neighbors to keep an eye on their care receiver or to leave a key for emergency situations. Thus, help was consistently offered, but rarely used.

## 4. Discussion: From Familiarism to Community?

This paper investigates changes in the organization of informal care during the COVID-19 pandemic, more specifically with respect to the role of the neighborhood in care provision versus family networks. Before beginning the interviews, we expected that the neighborhood would become a more important source of care, since the lockdown measures largely confined people to their neighborhood and prevented covering larger physical distances. This expectation was also formulated by other scholars, such as gerontologist Nico De Witte [46]. Despite the COVID-19 measures limiting people in travelling around, the distances in Flanders (and the whole of Belgium) remain relatively small in an international perspective. Several authors stress that distance is an important factor in receiving informal care [29,47]. Based on the European SHARE data (2004), Bonsang concluded that distance between parents and children is an important factor in receiving informal care [47]. In Belgium, the distance to the nearest child on average is around 22 km, while the European average is around 43 km [47], which might influence our results. 

Our main research question concerned the organization of informal care and potentially shifting responsibilities between familial and other informal caregivers due to the lockdown. Our core finding was that, especially during the lockdown’s first weeks, a shift could be detected towards more informal family care and help, as, in most cases, formal care and services were cancelled by the providing agencies. In others, the informal caregivers themselves cancelled the habitual formal caregiver(s) to protect their care receiver. In several cases, this led to higher care duties for themselves, which indicates that, in times of a health crisis, at least habitual caregivers would be willing to spend more time on informal care responsibilities. In these cases, movement restrictions were not seen as a major obstacle, but rather complicated the informal care provision. Similar results were found for Todorovic et al., who reported that lockdowns and other measures created extra complications to informal care provision [11]. In general, people’s dependence on the neighborhood and neighbors for help and care continued to be limited, both before and during the lockdown. Our findings suggest that neighborly care is not self-explanatory and hard to organize; several barriers need to be overcome, such as trusting the person offering help, not wanting to burden strangers, finding appropriate tasks, and so on. De Donder et al. [48] found that, during the first lockdown, those already enjoying neighborly support could count on increased support, while those who were the most vulnerable and disconnected were not reached, or did not respond to the offered help. Holton et al. [49] also reported trust between the older person and their befrienders as an essential prerequisite for their continued interaction during the pandemic. Although, during the lockdown, several initiatives and neighborhood networks were activated to help those in need, among our respondents, we did not find many people making use of them. However, we should note that our respondents were mostly family caregivers, while helping neighbors were rather exceptional in our sample. Caregivers’ perspectives might be different from those of older, care-receiving people, as caregivers might not be fully aware of smaller informal neighborly connections. Therefore, more research is needed about the coping strategies of older people themselves, particularly those without a family network, and what role the neighborhood plays in their cases. Nevertheless, other research confirms older people’s dismissive attitude, for example, towards help with groceries, which connotes independence [50].

Our findings clearly show that, according to their caregivers, there is little inclination among care receivers to appeal to non-family members (i.e., neighbors) for informal care. Even a health crisis does not evoke major change in this respect. This is in line with other studies reporting similar findings, such as Lightfoot et al. and Todorovic et al., who found that family members were reluctant to ask others out of fear of contagion [11,12]. It would be interesting for further research to investigate if this appeal to non-family members differs across neighborhoods dependent on, for example, the socioeconomic position of the inhabitants and spatial characteristics (e.g., dense working-class inner city neighborhoods vs. low-density suburban or rural villa parks). For now, and with a limited set of interviewees, we could not discover differences in that respect, while mentioning that even the inner city initiative reported a very low number of people appealing to them for help. 

At the same time, care receivers were reportedly reluctant to even ask other family members for help and care. Most of them grew up in a welfare state that stimulated people not to be dependent on family or friends for care needs, hence, asking for help might be seen as a failure [50]. Whereas the socialization of care fits into a neoliberal trend of reconfiguring the welfare state by encouraging people to depend more on their family and community, this trend is not yet fully integrated in people’s attitudes [10,51]. Our research indeed indicates that this change in culture and behavior does not take place automatically. Whereas our findings suggest that informal care by family members did intensify during the COVID-19 lockdown, this was not the case for community help and care (i.e., networks of volunteers organized by municipalities or otherwise). However, Sciensano’s report tells a different story: informal care actually decreased more than it increased during the first four weeks of lockdown [7]. Several explanations may exist for this apparent incongruity. In several cases, only one primary caregiver remained, and the others stopped providing care; travel restrictions may have stopped family members from visiting, neighbors may have worried about transmitting the virus, and so on. The former is in line with Lightfoot et al., who found that some of their respondents who previously divided caring responsibilities with others redistributed them at the beginning of the pandemic [12]. Thereby, some informal caregivers likely decreased their care load. As we noted before, Sciensano’s report does not differentiate between neighborly, friendly, or family help [7]. As we focus on the increase in informal family caregiving, this might explain why our results are not in line with the decreasing trend in some other reports. 

## 5. Conclusions 

This article aimed to better understand how informal caregivers coped with their caregiving duties in the context of the COVID-19 pandemic. The original addition to the body of knowledge concerns the neighborhood networks’ roles during the pandemic, which continued to be minimal. Whereas the importance of the neighborhood might have increased in other countries, the opposite seems to be true in Flanders: close family became more important than before. This asks for more comparative research in the future. Our results indicate the tendency of habitual informal caregivers to take on a higher caregiving load in times of crisis, whereas we found no indications pointing towards an increase in neighborly support. These results could indicate that the socialization of care tends to increase family responsibility, and has a rather small effect on the neighborhood level, at least in crisis situations. This risk of familiarism could have far-reaching consequences for the emancipation of women, who still carry the majority of caring responsibilities [14]. The tendency we found to rely even more on family members and less on neighborhood communities especially raises a flag with respect to the potential collateral damage cause by the socialization of care policy. 

More studies on the importance of older people’s networks are required, both within and outside the neighborhood. These networks may have changed during the pandemic, as new ones may have emerged because of neighborhood initiatives, or because of the shifting care responsibilities. As we indicated before, informal caregivers may not have been fully aware of their care receiver’s neighborly contacts. Some may have been reluctant to highlight the neighborhood’s involvement, since this might reflect poorly on themselves, serving as evidence of their shortcomings in their caring tasks. Our findings thus evoke new questions and reflections. Moreover, more research is needed on how older people who have no or weak family ties cope, and what role different living environments potentially play. In general, further research on the social (and care) networks of community-dwelling older adults is needed, as well as on conditions in later phases of the pandemic. The minor role that the neighborhood or local informal care network plays in our sample suggests that the shared responsibility between the individual, the family, and the community needs to be conceptualized in a more careful way, since community care will not happen automatically, especially in the context of a health crisis. 

## Data Availability

The data that support the findings of this study are available from the corresponding authors, upon reasonable request.

## References

[1] Agentschap Zorg en Gezondheid Extra Maatregelen voor de Bescherming van Ouderen Tegen Coronavirus. https://www.zorg-en-gezondheid.be/extra-maatregelen-voor-de-bescherming-van-ouderen-tegen-coronavirus.

[2] Agentschap Zorg en Gezondheid Woonzorgcentra Sluiten Standaard de Deuren. https://www.zorg-en-gezondheid.be/woonzorgcentra-sluiten-standaard-de-deuren.

[3] Baert D. Mogen We Nu Wél Of Niet Op Familiebezoek? Als We Het Coronavirus “Bij Het Nekvel Willen Grijpen” Niet. 18 March 2020. VRT Nieuws. https://www.vrt.be/vrtnws/nl/2020/03/18/familiebezoek/.

[4] Hale B., Barrett P., Gauld R. (2010). The Age of Supported Independence: Voices of In-Home Care.

[5] Lightfoot E., Moone R. (2020). Caregiving in Times of Uncertainty: Helping Adult Children of Aging Parents Find Support during the COVID-19 Outbreak. J. Ger. Soc. Work.

[6] Mertens B. Leuven blijft inwoners helpen nu tweede golf van start is gegaan: Leuven Helpt wordt permanent platform voor hulp.Het Laatste Nieuws, 3 August 2020. https://www.hln.be/in-de-buurt/leuven/leuven-blijft-inwoners-helpen-nu-tweede-golf-van-start-is-gegaan-leuven-helpt-wordt-permanent-platform-voor-hulp~a32bf80e/.

[7] Sciensano E. (2020). COVID-19 Gezondheidsenquête: Eerste resultaten.

[8] Chan E.Y., Lo E.S., Huang Z., Kim J.H., Hung H., Hung K.K., Wong E.L., Wong S.Y., Gobat N. (2020). Characteristics and well-being of urban informal home care providers during COVID-19 pandemic: A population-based study. BMJ Open.

[9] Budnick A., Hering C., Eggert S., Teubner C., Suhr R., Kuhlmey A., Gellert P. (2021). Informal Caregivers during the COVID-19 Pandemic Perceive Additional Burden: Findings from an Ad-Hoc Survey in Germany. BMC Health Serv. Res..

[10] Rodrigues R., Simmons C., Schmidt A.E., Steiber N. (2021). Care in times of COVID-19: The impact of the pandemic on informal caregiving in Austria. Eur. J. Ageing.

[11] Todorovic N., Vracevic M., Rajovic N., Pavlovic V., Madzarevic P., Cumic J., Mostic T., Milic N., Rajovic T., Sapic R. (2020). Quality of Life of Informal Caregivers behind the Scene of the COVID-19 Epidemic in Serbia. Medicina.

[12] Lightfoot E., Moone R., Suleiman K., Otis J., Yun H., Kutzler C., Turck K. (2021). Concerns of Family Caregivers during COVID-19: The Concerns of Caregivers and the Surprising Silver Linings. J. Gerontol. Soc. Work..

[13] Fret B., Mondelaers B., De Donder L., Switsers L., Smetcoren A.-S., Verté D., The D-SCOPE consortium (2018). Exploring the Cost of ‘Ageing in Place’: Expenditures of Community-Dwelling Older Adults in Belgium. Ageing Int..

[14] De Tavernier W., Draulans V. (2019). Negotiating informal elder care, migration and exclusion: The case of a Turkish immigrant community in Belgium. Int. J. Ageing Later Life.

[15] Alders P., Schut F. (2019). The 2015 Long-Term Care Reform in the Netherlands: Getting the Financial Incentives Right?. Health Pol..

[16] Morikawa M. (2014). Towards community-based integrated care: Trends and issues in Japan’s long-term care policy. Int. J. Integr. Care.

[17] Fernández-Carro C. (2016). Ageing at home, co-residence or institutionalisation? Preferred care and residential arrangements of older adults in Spain. Ageing Soc..

[18] Löfqvist C., Granbom M.M., Himmelsbach I., Iwarsson S., Oswald F., Haak M. (2013). Voices on Relocation and Aging in Place in Very Old Age—A Complex and Ambivalent Matter. Gerontologist.

[19] Pannecoucke I., De Decker P. (2017). Woonsituatie En-Dynamieken Bij Ouderen: Blijven of Verhuizen? Leuven: Steunpunt Wonen. https://steunpuntwonen.be/Documenten_2016-2020/Onderzoek_Werkpakketten/WP_6_Verhuisbewegingen_en_ruimtelijke_dynamiek_op_de_woningmarkt/WP6a_1_RAPPORT.

[20] Horner B., Boldy D.P. (2008). The benefit and burden of “ageing-in-place” in an aged care community. Aust. Health Rev..

[21] Means R. (2007). Safe as Houses? Ageing in Place and Vulnerable Older People in the UK. Soc. Policy Adm..

[22] Bakx P., Wouterse B., Van Doorslaer E., Wong A. (2020). Better off at home? Effects of nursing home eligibility on costs, hospitalizations and survival. J. Health Econ..

[23] Golant S.M. (2015). Aging in the Right Place.

[24] Oswald F., Jopp D., Rott C., Wahl H. (2010). Is Aging in Place a Resource for or Risk to Life Satisfaction?. Gerontologist.

[25] Sun Y., Phillips D.R., Wong M. (2018). A study of housing typology and perceived age-friendliness in an established Hong Kong new town: A person-environment perspective. Geoforum.

[26] Lord S., Luxembourg N. (2007). The Mobility of elderly Residents Living in Suburban Territories. JHFTE.

[27] Greenfield E.A., Black K., Buffel T., Yeh J. (2019). Community Gerontology: A Framework for Research, Policy, and Practice on Communities and Aging. Gerontologist.

[28] Kelley J., Dannefer D., Masarweh L., Buffel T., Handler S., Phillipson C. (2018). Addressing Erasure, Microfication and Social Change. Age-Friendly Cities and Communities: A Global Perspective.

[29] Broese van Groenou M.I., De Boer A. (2016). Providing informal care in a changing society. Eur. J. Ageing.

[30] Robine J.M., Michel J.P., Herrmann F.R. (2007). Who will care for the oldest people in our ageing society?. BMJ.

[31] Hermann F.R., Michel J.P., Robine J.M. (2010). Worldwide decline in the oldest old support ratio. Eur. Geriatr. Med..

[32] Heitink E., Heerkens Y., Engels J. (2017). Informal care, employment and quality of life: Barriers and facilitators to combining informal care and work participation for healthcare professionals. Work.

[33] Moussa M.M. (2019). The relationship between elder care-giving and labour force participation in the context of policies addressing population ageing: A review of empirical studies published between 2006 and 2016. Ageing Soc..

[34] Haberkern K., Szydlik M. (2010). State care provision, societal opinion and children’s care of older parents in 11 european countries. Ageing Soc..

[35] Kromhout M., De Klerk M., Kornalijnslijper N. (2018). Veranderde Zorg en Ondersteuning Voor Mensen Met een Beperking.

[36] I&O Research (2019). De YEP Van Tegenwoordig: De Toekomst Van Nieuwe Ouderen. Onderzoek Onder 55-Tot 75-Jarigen in Opdracht Van TROUW.

[37] Carmichael F., Charles S., Hulme C. (2010). Who will care? Employment participation and willingness to supply informal care. J. Health Econ..

[38] Hassink W.H.J., Van den Berg B. (2011). Time-bound opportunity costs of informal care: Consequences for access to professional care, caregiver support, and labour supply estimates. Soc. Sci. Med..

[39] Prevo L., Linssen E., Hajema K., Kremers S., Crutzen R., Schneider F. (2017). Exploring Informal Caregivers’ Views on Their Perceived Burden. Home Health Care Manag. Pract..

[40] Greenwoon N., Pound C., Brearley S., Smith R. (2019). A qualitative study of older informal carers’ experiences and perceptions of their caring role. Maturitas.

[41] Braun V., Clarke V. (2006). Using thematic analysis in psychology. Qual. Res. Psychol..

[42] Maguire M., Delahunt B. (2017). Doing a Thematic Analysis: A Practical, Step-By-Step Guide for Learning and Teaching Scholars. Irel. J. High. Educ..

[43] Nowell L.S., Norris J.M., White D.E., Moules N.J. (2017). Thematic Analysis. Int. J. Qual. Methods.

[44] Grenzen A.Z. (2020). Overgelaten Aan Hun Lot. De Ervaring Van Artsen Zonder Grenzen in De Woonzorgcentra Tijdens De Covid-19-Epidemie in België.

[45] Amnesty International (2020). Woonzorgcentra in De Dode Hoek. Mensenrechten Van Ouderen Tijdens De Covid-19-Pandemie in België.

[46] De Witte N. Je Zit Tussen Vier Muren, Je Zit Gevangen: Het Eenzaamheidsvirus Flakkert Op Bij Ouderen Door Corona. News Interview 29 September 2020. https://www.vrt.be/vrtnws/nl/2020/09/29/terzake-eenzaamheid/.

[47] Bonsang E. (2009). Does informal care from children to their elderly parents substitute for formal care in Europe?. J. Health Econ..

[48] De Donder L., Hoens S., Kint O., Smetcoren A.-S., Stegen H. (2021). Lokaal Samenwerken in Zorgzame Buurten. Koning Boudewijn Stichting: Brussel, Belgium. https://www.kbs-frb.be/nl/Activities/Publications/2021/20210413PP?utm_source=newsletter&hq_e=el&hq_m=6377299&hq_l=4&hq_v=09cd12403a.

[49] Holton E., Fitzpatrick R., Maguire R., Commins S., Scharf T., Lawlor B., Johnson N., Hannigan C., McHugh Power J. (2021). Older Users of a Befriending Service in Ireland and the Maintenance of Personal Autonomy during the COVID-19 Pandemic. Int. J. Environ. Res. Public Health.

[50] Bredewold F., Verplanke L., Kampen T., Tonkens E., Duyvendak J.W. (2020). The care receivers perspective: How care-dependent people struggle with accepting help from family members, friends and neighbours. Health Soc. Care Community.

[51] Duyvendak J.W. (2017). Thuis. Het Drama Van Een Sentimentele Samenleving.

